# Analgesic and Anti-Inflammatory Activities of the Methanol Extract from *Pogostemon cablin*

**DOI:** 10.1093/ecam/nep183

**Published:** 2011-02-14

**Authors:** Tsung-Chun Lu, Jung-Chun Liao, Tai-Hung Huang, Ying-Chih Lin, Chia-Yu Liu, Yung-jia Chiu, Wen-Huang Peng

**Affiliations:** ^1^Graduate Institute of Chinese Pharmaceutical Sciences, College of Pharmacy, China Medical University, 91, Hsieh Shih Road, Taichung, Taiwan; ^2^School of Pharmacy, College of Pharmacy, China Medical University, 91, Hsieh Shih Road, Taichung, Taiwan; ^3^Department of Optometry, Jen-Teh Junior College of Medicine, Nursing and Management, No. 79-9, Sha-Luen-Hu, Xi Zhou Li, Hou-Loung Town, Miaoli County 35664, Taiwan; ^4^Department of Nursing, Jen-Teh Junior College of Medicine, Nursing and Management, No. 79-9, Sha-Luen-Hu, Xi Zhou Li, Hou-Loung Town, Miaoli County 35664, Taiwan

## Abstract

*Pogostemon cablin* (PC) is a herbal medicine traditionally applied to treat not only common cold, nausea and diarrhea but also headache and fever. The aim of this study was to investigate the analgesic and anti-inflammatory properties of standardized PC methanol extract (PCMeOH) *in vivo*. Investigations were performed in mice with two analgesic models. One was acetic acid-induced writhing response and the other formalin-induced paw licking. The anti-inflammatory effect was tested by *λ*-carrageenan (Carr)-induced mice paw edema. These analgesic experimental results indicated that PCMeOH (1.0 g/kg) decreased the acetic acid-induced writhing responses and PCMeOH (0.5 and 1.0 g/kg) decreased the licking time in the second phase of the formalin test. Moreover, Carr-induced paw edema inflammation was significantly reduced in a dose-dependent manner when PCMeOH (0.5 and 1.0 g/kg) was administered 3 and 4 h after the Carr injection. Mechanistic studies showed that PCMeOH decreased the levels of malondialdehyde in the edema paw by increasing the activities of anti-oxidant enzymes, such as superoxide dismutase, glutathione peroxidase and glutathione reductase, in the liver and decreasing the cyclooxygenase 2 and tumor necrosis factor-*α* activities in the edema paw. This study has demonstrated the analgesic and anti-inflammatory effects of PCMeOH, thus verifying its popular use in traditional medicine.

## 1. Introduction

Cerebral and coronary artery diseases are the leading causes of death around the world. More and more researchers have demonstrated that abnormal inflammatory cells form a plaque and play an essential role in the pathogenesis and progression of atherosclerosis [1]. Anti-inflammatory agents have been shown to have good effects on the prevention and treatment of atherosclerosis and coronary artery diseases [2]. Therefore, developing novel anti-inflammatory drugs is very important today.

Analgesic drugs operate in different ways on the peripheral and central nervous systems. Narcotic drugs, such as morphine, have analgesic activity, which inhibits the delivery of pain impulses [3, 4]. Peripheral drugs include paracetamol and non-steroidal anti-inflammatory drugs (NSAIDs). Only NSAIDs possess analgesic and anti-inflammation activity due to the mechanism of inhibiting cyclooxygenases (COXs) for the decrease in prostaglandin (PG) production, which consequently reduces pain and inflammation. However, the NSAIDs used clinically are often of limited application because of their common side effects, such as gastrointestinal (GI) hemorrhage [5]. As to COX, COX-1 is thought to provide cytoprotection, whereas COX-2 inhibitor may have selective anti-inflammatory properties and lack GI side effects [6].

PC is native to tropical regions of Asia and now cultivated extensively in Caribbean countries, China, India, Indonesia, Malaysia, Mauritius, Philippines, West Africa and Vietnam. Its dried aerial part is commonly known as “Guang-Huo-Xiang" [7]. Several studies have been performed on the composition of PC and presence of patchouli alcohol, pogostone, eugenol, *α*-bulnesene, rosmarinic acid and others [8]. Essential oils constitute *∼*1.5% of PC, among which >50% is patchouli alcohol. The alcoholic extract of PC contains the major active ingredient [9]. PC is used by traditional physicians in treating not only the common cold, nausea and diarrhea but also headaches and fever [10]. The volatile oil of PC has been widely used in cosmetics and oral hygiene products, such as scent perfumes and flavor toothpaste. Pharmacological activities of this oil have been demonstrated repeatedly in modern research, including its anti-emetic, anti-bacterial and anti-fungal activities [11–13]. PC contains sesquiterpenes cytotoxic chalcones [14] and anti-mutagenic flavones. However, there is little research regarding the information on the analgesic and anti-in*ﬂ*ammatory activities of PC *in vivo* and *in vitro*.

This study investigated the anti-inflammatory and analgesic activities of PC methanol extract (PCMeOH). Its analgesic activity was evaluated using the acetic acid-induced writhing response and formalin test, because acetic acid-induced abdominal constriction was associated with the involvement of peripheral mechanisms [15]. The formalin test was performed according to the method of Dubuisson and Dennis [16]. The time spent in behavioral responses to nociception, including biting, licking and scratching of the injected paw, was noted. The test conducted involves a biphasic response with the early phase, the direct effect of formalin on nociceptors and the late phase due to inflammation [17].

The anti-inflammatory activity of PCMeOH was determined using the Carr-induced paw edema model, which is a useful model to assess inflammation. The model has been correlated with the increased production of PGs and, more recently, has been attributed to the induction of inducible COX-2 in the hind paw [18]. The Carr-induced inflammatory response has been linked to neutrophil infiltration and the production of neutrophil-derived free radicals, such as hydrogen peroxide (H_2_O_2_), superoxide (O_3_^−^) and hydroxyl radicals (OH^−^), as well as to the release of other neutrophil-derived mediators [19]. Superoxide dismutase (SOD), the first line of defense in the body against O_2_, is considered the most effective anti-oxidant [20]. Glutathione peroxidase (GPx), which accelerates the reduction of H_2_O_2_ or other organic hydroperoxides (ROOH) in the presence of GSH, serves as a second line of defense against hydroperoxides [21]. Glutathione reductase (GRx) plays a crucial role in cellular defense against oxidative stress by preventing accumulation of GSSG, thus maintaining the redox state [22]. This study also observed tumor necrosis factor-*α* (TNF-*α*) level and COX-2 protein expression in the edema paw and activities of anti-oxidant enzymes in the liver. Indomethacin (Indo) was used as a positive control.

## 2. Materials and Methods

### 2.1. Plant Material

The plants of *Pogostemon cablin* (Blanco) Benth (Family Lamiaceae) were collected from Taichung, Taiwan, in July 2006 and identified by Dr Chao-Lin Kuo, leader of the School of Chinese Medicine Resources (SCMR). The voucher specimen (Number: CMU MO 0720) was deposited at SCMR.

### 2.2. Preparation of Plant Extract

Dried PC (1800 g), made from the aerial part and leaves of plants, was sliced into small pieces and ground into a powder. Ten liters of methanol was added to the dried powder and extracted for 24 h per cycle four times. The extracts were filtered, combined and concentrated under reduced pressure at 40°C to obtain the PCMeOH extract. The yield ratio of the PCMeOH extract (53.1 g) was 2.95%.

### 2.3. Animals

Each experimental group consisted of eight Imprinting Control Region (ICR) mice (18–22 g). They were obtained from the National Laboratory Animal Breeding and Research Center, National Science Council, Taiwan, and housed in standard cages at a constant temperature of 22 ± 1°C and relative humidity of 55 ± 5% with 12-h dark–light cycles (08 : 00–20 : 00) for at least 1 week before the experiment. They were fed with food and water *ad libitum*. The experimental protocol was approved by the Committee on Animal Research, China Medical University. The minimum number of animals and duration of observations required to obtain consistent data were used.

### 2.4. Chemicals

*λ*-Carrageenan, indomethacin, glycine, phosphate-buffered saline (PBS) solution and glutaraldehyde were purchased from Sigma-Aldrich, Inc. (St Louis, MO, USA). Formalin was purchased from Nihon Shiyaku Industry Ltd, Japan; the activity assay kits, from Randox Laboratory Ltd; TNF-*α*, from Biosource International, Inc., and COX-2 anti-body (ab16701), from Abcam plc.

### 2.5. Acetic Acid-Induced Writhing Response

The writhing test in mice was carried out using the method proposed by Lu [23]. The writhes were induced by intraperitoneal (i.p.) injection of 1.0% acetic acid (v/v, 0.1 mL/10 g body weight). Three different doses (0.1, 0.5 and 1.0 g/kg) of PCMeOH were administered orally to each group of mice 60 min before acetic acid injection. Indo as a positive control was administered 30 min prior to chemical stimulus. The data were recorded 5 min after acetic acid injection. The number of muscular contractions was counted over a period of 10 min after acetic acid injection. The data collected would represent the total number of writhes observed during the 10-min period.

### 2.6. Formalin Test

The method used in our study was similar to that described in the previous study [24]. Pain was induced by injecting 20 *μ*L of 5% formalin in distilled water in the subplantar of the right hind paw. PCMeOH (0.1, 0.5 and 1.0 g/kg, p.o.) was administered 60 min before formalin injection. Indo (10 mg/kg, p.o.) was administered 30 min before formalin injection. The control group received the same volume of saline by oral administration. These mice were individually placed in a transparent Plexiglas cage (25 × 15 × 15 cm). The time spent for licking and biting the injected paw, as the indicators of pain, was recorded separately at 0–5 min (first phase or neurogenic pain) and 20–30 min (second phase or inflammatory pain) [17].

### 2.7. Carr-Induced Mice Paw Edema

The anti-inflammatory activity of PCMeOH was determined by the Carr-induced edema test in the hind paws of mice. Male ICR mice (eight per group) were fasted for 24 h before the experiment, with free access to water. Fifty microliters of 1% Carr suspension in saline was injected into the plantar side of the right hind paws of the mice [25]. Paw volume was measured immediately at 1, 2, 3 and 4 h after the administration of the Carr using a plethysmometer. The degree of swelling was evaluated by the delta volume (*a* − *b*), where *a* and *b* are the volume of the right hind paw after and before the Carr treatment, respectively. Indo was used as a positive control compound [26], which was administered intraperitoneally (i.p.) 150 min after Carr injection. PCMeOH was administered orally 120 min after Carr injection.

In the secondary experiment, the whole right hind paw and liver tissues were taken at the third hour. The right hind paw tissue was rinsed in ice-cold normal saline and immediately placed in cold normal saline four times their volume and finally homogenized at 4°C. Then, the homogenate was centrifuged at 11,270 g for 5 min. The supernatant was obtained and stored at −80°C for the TNF-*α*, COX-2 and malondialdehyde (MDA) assays.

On the other hand, the whole liver tissue was rinsed in ice-cold normal saline and immediately placed in cold normal saline of the same volume and finally homogenized at 4°C. Then, the homogenate was centrifuged at 11,270 g for 5 min. The supernatant was obtained and stored at −80°C for the anti-oxidant enzyme (SOD, GPx and GRx) activity assays.

#### 2.7.1. MDA Assay

MDA was evaluated by the thiobarbituric acid-reacting substance (TRARS) method [27]. First, the paw tissues were homogenized in buffered saline (1 : 4); then, 400 *μ*L of 1,1,3,3-tetraethoxypropan trichloroacetic acid (28% w/v) was added to 200 *μ*L of this mixture and centrifuged in 3000 g for 30 min. After that, 300 *μ*L of the supernatant was added to 150 *μ*L of 2-thiobarbituric acid (1% w/v). The mixture was incubated for 45 min in a boiling water bath, and then 450 *μ*L n-butanol was added; the solution was centrifuged and cooled, and absorption of the supernatant was recorded in 532 nm by the microplate reader (VersaMax, Massachusetts, USA). Tetramethoxypropane was used as standard. MDA levels were expressed as nanomoles per milligram of protein. Protein concentration was measured by Lowry method [28]. Bovine serum albumin was used as standard.

#### 2.7.2. Anti-Oxidant Enzymes' Activities

The following biochemical parameters were analyzed to detect the anti-oxidant activities of PC by the methods described below. SOD enzyme activity was determined at room temperature according to the method of Misra and Fridovich [29]. One hundred microliters of tissue extract was added to 880 *μ*L (pH 10.2, 0.1 mM EDTA) of carbonate buffer. Twenty microliters of 30 mM epinephrine (in 0.05% acetic acid) was added to the mixture at 480 nm for 4 min on a Hitachi U 2000 Spectrophotometer. The enzyme activity was expressed as the amount of enzyme that inhibits the oxidation of epinephrine by 50%, which is equal to 1 unit.

GPx enzyme activity was determined according to the method of Flohe and Gunzler [30] at 37°C. The reaction mixture composed of 500 *μ*L phosphate buffer, 100 *μ*L 0.01 M GSH (reduced form), 100 *μ*L 1.5 mM NADPH and 100 *μ*L GRx (0.24 units). One hundred microliters of the tissue extract was added to the reaction mixture and incubated at 37°C for 10 min. Then, 50 *μ*L of 12 mM *t*-butyl hydroperoxide was added to 450 *μ*L of tissue reaction mixture and measured at 340 nm for 180 s. The molar extinction coefficient of 6.22 × 10^−3^ was used to determine the enzyme activity. One unit of activity is equal to the millimolar of NADPH oxidized per minute per milligram of protein.

GRx enzyme activity was determined following the method of Carlberg and Mannervik [31] at 37°C. Fifty microliters of NADPH (2 mM) in 10 mM Tris buffer (pH 7.0) was added in the cuvette containing 50 *μ*L of GSSG (20 mM) in phosphate buffer. One hundred microliters of tissue extract was added to the NADPH-GSSG-buffered solution and measured at 340 nm for 3 min. The molar extinction coefficient of 6.22 × 10^−3^ was used to determine GRx enzyme activity. One unit of activity is equal to the millimolar of NADPH oxidized per minute per milligram of protein.

#### 2.7.3. Tissue COX-2 by Quartz Crystal Microbalance

The P-sensor 2000 designed by ANT is based on the principle of piezoelectric biosensor. It is made of three portions including electronic oscillation circuit, frequency counter and piezoelectric quartz of fixed biosensor molecule (p-chip). The piezoelectric quartz crystal consists of a quartz crystal slab with a layer of gold electrode on each side. It is the signal conversion component of the piezoelectric sensor chip and can convert the result sensed by the sensor molecule into electronic signal to be amplified. The function of gold electrodes is mainly to introduce an oscillating electric field perpendicular to the surface of the chip so that the internal part of the chip generates mechanical oscillation because of the piezoelectric effect. If the thickness of the quartz crystal is fixed, the mechanical oscillation will be generated at a fixed frequency. Using a suitable electronic oscillation circuit, the resonant frequency can be measured.

P-sensor 2000 (Asia New Technology, Taiwan), based on quartz crystal microbalance (QCM), was used to monitor the antibody-antigen interaction in real time [32]. The PBS, a mobile carrier, would flow through the sensor cell with the antibody-immobilized chip in flow rate of 30 *μ*L/min and clean the fluid lines of QCM, alternating with the 1 N NaOH and 1 N HCl solution and ultra-pure water before the measurement. After the introduction of PBS to fill the sensor cell, the frequency shift of QCM reached a steady equilibrium (“ΔF" < 0.2 Hz/min) and was defined as a zero baseline “F0". Upon the injection of experimental solution into the sensor cell, the dynamic interactions between antigens and immobilized antibodies were monitored, and the frequency shifts were recorded for the next steady equilibrium, “F". Thus, the apparent frequency change of crystal oscillator, “ΔF", can be measured by subtracting “F" from “F0". All of PBS and diluted sera solutions were filtered with Millex GP filter unit (0.22 *μ*m, PES membrane; Millipore, Ireland) and degassed before use. The sensor chips were disposable to ensure the sensitivity and reproducibility of each of the QCM experiments. With a temperature controller, the temperature of the sensor cell was controlled at a constant temperature of 25.0 (±0.1)°C to suppress the fluctuations of kinetics by ambient environment.

### 2.8. Tissue TNF-*α* by ELISA

Tissue levels of TNF-*α* were determined using a commercially available enzyme-linked immunosorbent assay (ELISA) kit according to the manufacturer's instruction. The measurements were performed according to the manufacturer's protocols. The absorbance at 450 and 540 nm was measured on a microplate reader (VersaMax, Massachusetts, USA). TNF-*α* was determined from a standard curve for the combination of these cytokines [33].

#### 2.8.1. Statistical Analysis

All the data were expressed as mean ± SEM. Statistical analysis was carried out using one-way analysis of variance (ANOVA), followed by Scheffe's multiple range test. The criterion for statistical significance was *P* < .05.

## 3. Results

### 3.1. Acetic Acid-Induced Writhing Response

**Figure 1:**
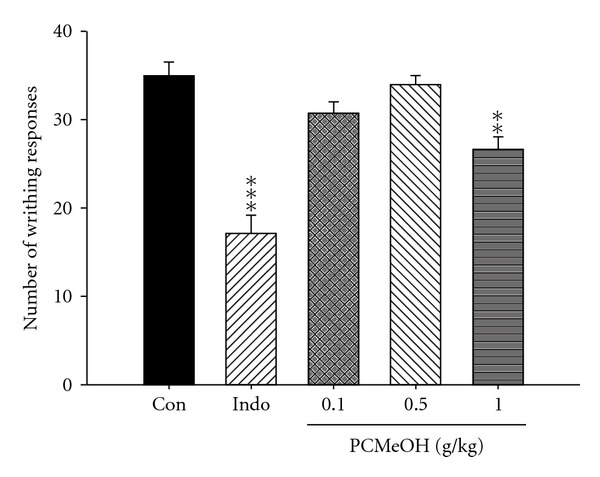
Analgesic effect of the PCMeOH and Indo on acetic acid-induced writhing response in mice. Each value represents mean ± SEM. **P < .01, ***P < .001 as compared with the control (Con) group. (One-way ANOVA followed by Scheffe's multiple range test).

The results of acetic acid-induced writhing responses in mice that indicate the analgesic activity of the methanol extracts of PCMeOH are presented in Figure 1. It was found that the PCMeOH (1.0 g/kg) and Indo (10 mg/kg) showed inhibition in this model (*P* < .01–.001). 

### 3.2. Formalin Test

**Figure 2:**
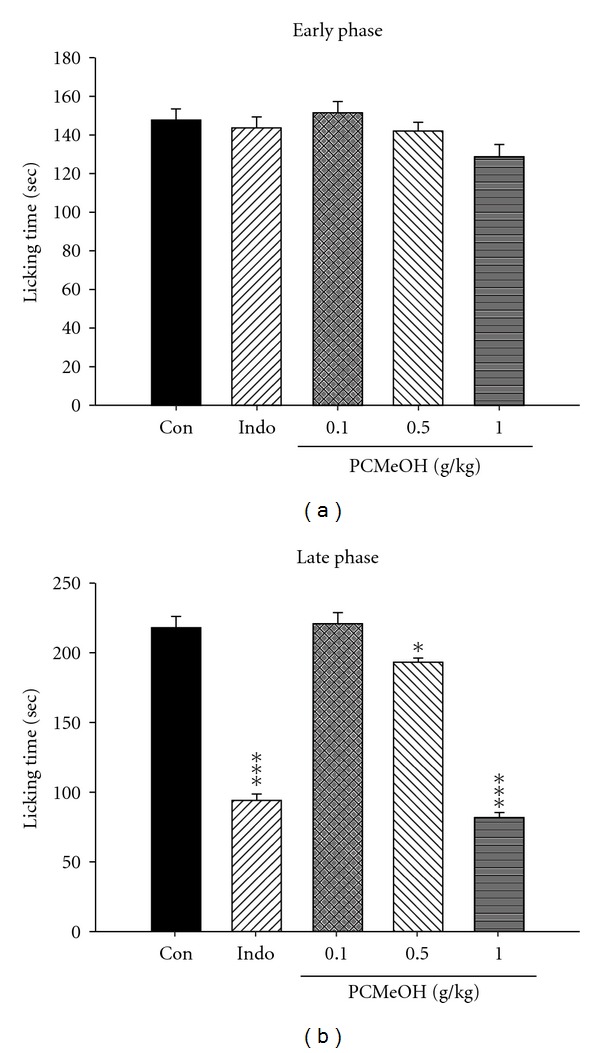
Analgesic effect of the PCMeOH and Indo on the (a) early phase and (b) late phase in formalin test in mice. Each value represents mean ± SEM. *P < .05, ***P < .001 as compared with the control (Con) group. (One-way ANOVA followed by Scheffe's multiple range test).

PCMeOH demonstrated a dose-dependent relationship in both phases of formalin-induced pain. In the early phase, there are no significant inhibitions with the dose of PCMeOH (0.1, 0.5 and 1.0 g/kg) and Indo (10 mg/kg) compared with the control group (Figure 2(a)). In the late phase, the dose of PCMeOH (0.5 and 1.0 g/kg) and Indo (10 mg/kg) significantly reduced the nociception (*P* < .05–.001; Figure 2(b)). 

### 3.3. Carr-Induced Mice Paw Edema

**Figure 3:**
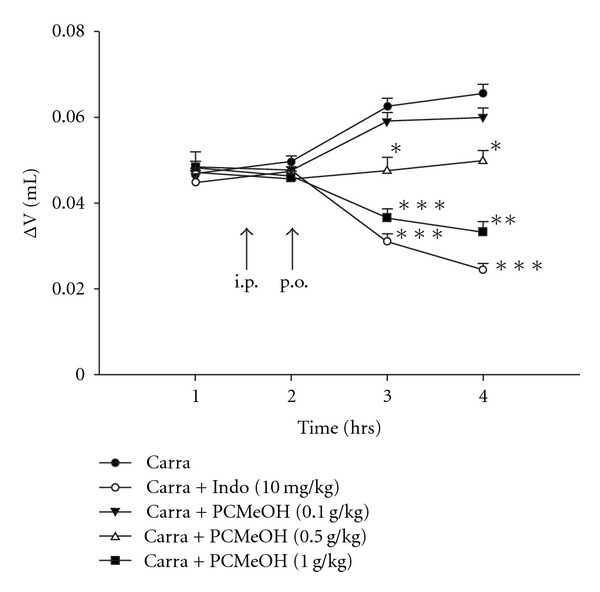
Effect of the PCMeOH and Indo on hind paw edema induced by Carr in mice. Indo was administered i.p. at 150 min after Carr injection. PCMeOH was administered orally (p.o.) at 120 min after Carr injection. Each value represents mean ± SEM. *P < .05, **P < .01, ***P < .001 as compared with the Carr group. (One-way ANOVA followed by Scheffe's multiple range test).

PCMeOH (0.5 and 1.0 g/kg) was observed to inhibit (*P* < .05–.001) the development of paw edema induced by Carr after 3 and 4 h of treatment. Indo (10 mg/kg) significantly decreased the Carr-induced paw edema after 3 and 4 h of treatment (*P* < .001; Figure 3). 

#### 3.3.1. MDA Level

**Figure 4:**
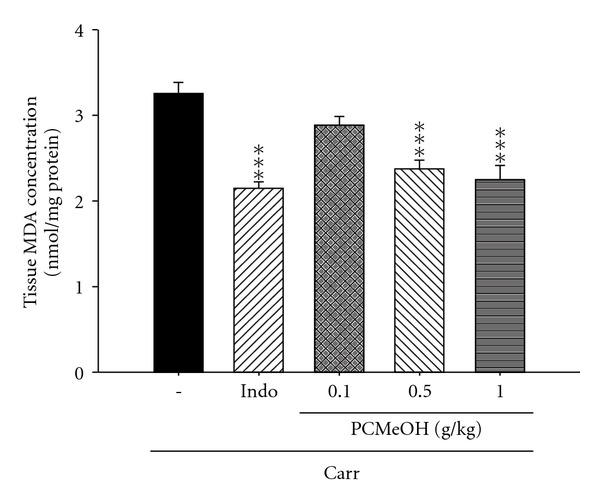
The MDA accumulation of the PCMeOH and Indo at the third hour after Carr injection in the mice edema paw. Each value represents mean ± SEM. ***P < .001 as compared with the Carr group. (One-way ANOVA followed by Scheffe's multiple range test).

In the control group, MDA level in the edema paw induced by Carr increased significantly. However, the MDA level decreased significantly with treatment of PCMeOH (0.5 and 1.0 g/kg), as well as 10 mg/kg Indo (*P* < .001; Figure 4). 

#### 3.3.2. The Activities of Anti-Oxidant Enzymes

**Figure 5:**
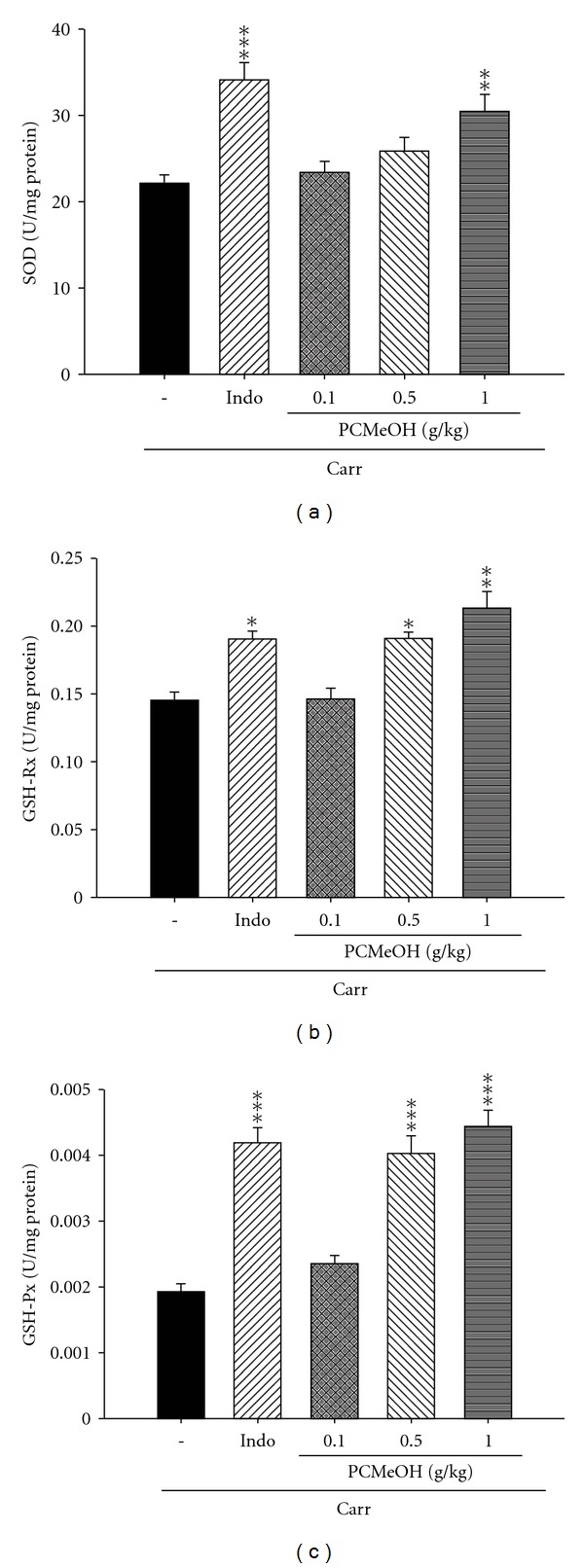
Effect of the PCMeOH and Indo on the liver (a) SOD, (b) GRx and (c) GPx activities in mice. Each value represents mean ± SEM. *P < .05, **P < .01, ***P < .001 as compared with the Carr group. (One-way ANOVA followed by Scheffe's multiple range test).

At the third hour following the intrapaw injection of Carr, liver tissues were also analyzed for the biochemical parameters, such as SOD, GPx and GRx activities. SOD activity in the liver tissue decreased significantly by Carr administration. SOD activity increased significantly after treatment with 10 mg/kg Indo (*P* < .001; Figure 5(a)). Carr administration markedly decreased GPx and GRx activities in the liver tissues. GRx activities in the liver tissues increased significantly with the treatment of PCMeOH (0.5 and 1.0 g/kg) and Indo (10 mg/kg) (*P* < .01–.001; Figure 5(b)). GPx activities in the liver tissues increased significantly with the treatment of PCMeOH (0.5 and 1.0 g/kg) and Indo (10 mg/kg) (*P* < .001; Figure 5(c)). 

#### 3.3.3. COX-2 Level

**Figure 6:**
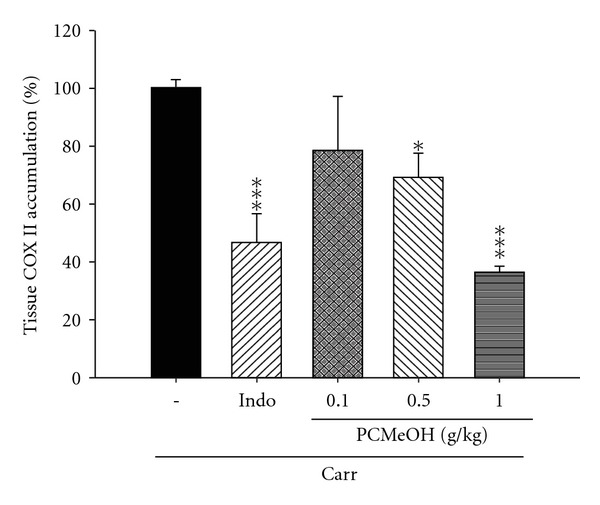
The COX-2 accumulation of the PCMeOH and Indo at the third hour after Carr injection in the mice edema paw. All values represent means ± SEM (*n* = 10). *P < .05, ***P < .001 as compared with the Carr group. (One-way ANOVA followed by Scheffe's multiple range test).

The activity of COX-2 increased significantly at the edema paw of mice after Carr administration for the third hour. COX-2 activity was inhibited significantly by treatment with PCMeOH (0.5 and 1.0 g/kg) or Indo (10 mg/kg) (*P* < .05–.001; Figure 6). 

#### 3.3.4. TNF-*α* Level

**Figure 7:**
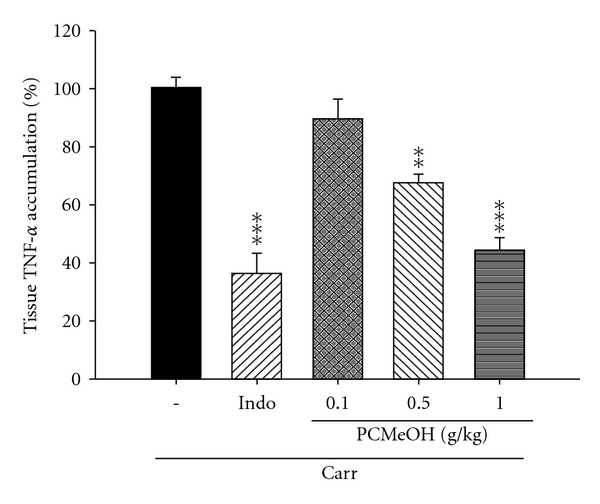
The TNF-*α* accumulation of the PCMeOH and Indo at third hour after Carr injection in the mice edema paw. All values represent as means ± SEM (*n* = 10). **P < .01, ***P < .001 as compared with the Carr group. (One-way ANOVA followed by Scheffe's multiple range test).

The level of TNF-*α* increased significantly at the edema paw of mice after Carr administration for 3 h. At the third hour, TNF-*α* level was inhibited significantly after treatment with PCMeOH (0.5 and 1.0 g/kg) or Indo (10 mg/kg) (*P* < .01–.001; Figure 7). 

## 4. Discussion

We have evaluated the putative analgesic and anti-inflammatory activities of PCMeOH to demonstrate its pain- and inflammation-relieving effects. Two different analgesic testing methods were used with the objective of identifying possible peripheral and central effects of the test substances. In the acetic acid-induced writhing response, the visceral pain model, the analgesic mechanism of abdominal writhing was induced by acetic acid, which involves the release of arachidonic acid (AA) via COX and PG biosynthesis [34]. PCMeOH at the oral dose of 1.0 g/kg significantly decreased the writhing response of acetic acid-induced mice. In addition, the formalin test involved a biphasic response: the direct effect of formalin on nociceptors in the early phase and inflammation in the late phase [35, 36]. PCMeOH was administered orally (0.5 and 1.0 g/kg) to the mice, which produced dose-related inhibition late-phase pain but did not inhibit neurogenic (early phase) pain caused by intraplantar injection of formalin. These results suggested that PCMeOH possessed significant analgesic effect. The analgesic effect may be due to anti-inflammatory effect.

The Carr-induced inflammation was a standard model of screening for anti-inflammatory activity in various experimental compounds [37]. Carr-induced edema is characterized by the presence of PGs and other compounds of slow reaction [38]. COX-2 is an inducible isoform found in activated inflammatory cells that generates prostanoid mediators of inflammation [39]. Inhibition of COX-2 protein expression has also become the most popular target for screening anti-inflammatory agents and the study of pathogenesis and pathology of the inflammatory and nociceptive processes in animal models [40]. TNF-*α* is a major mediator in inflammatory responses, inducing innate immune responses by activating T cells and macrophages and stimulating the secretion of other inflammatory cytokines [41]. As cytokines are critical to the pathogenesis of inflammatory disorders, inhibition of their production and action can provide therapeutic benefits. Previous studies have shown significant correlations among cytokine production, COX-2 protein expression and PG synthesis in the paw tissues of mice in which edema was invoked by intraplantar injection of Carr [42]. PCMeOH at dosages of 0.5–1.0 g/kg significantly suppressed the protein expression of COX-2 level in the edema paw tissues of mice. The production of multiple proinflammatory cytokines, such as TNF-*α*, in edema paw tissues of mice was also decreased by PCMeOH treatment. These results suggest that PCMeOH played a role in the anti-inflammatory activities in the model of Carr-induced paw edema of mice through the inhibition of TNF-*α* and COX-2 level.

The Carr-induced inflammatory response has been linked to neutrophil infiltration and the production of neutrophil-derived free radicals, such as hydrogen peroxide, superoxide and hydroxyl radicals, as well as to the release of other neutrophil-derived mediators [19]. Some studies demonstrate that the inflammatory effect induced by Carr is associated with free radicals. Free radicals and PG are released when Carr is administered for 1–6 h [43]. Also, the paw edema was raised to maximum at the third hour [44]. Research has demonstrated that MDA production is caused by free radicals attacking plasma membranes [45–47]. Thus, Carr-induced inflammatory effect results in the accumulation of MDA [48]. Glutathione is a known oxyradical scavenger. The enhancement of glutathione levels reduces MDA production. Cuzzocrea suggests that endogenous glutathione plays an important role against Carr-induced local inflammation [49]. PCMeOH and Indo showed anti-inflammatory activity in Carr-induced mice paw edema at the third and fourth hours. PCMeOH significantly increased the SOD, GRx and GPx activities (Figure 5) and significantly decreased the MDA level (Figure 4). We assume that the suppression of MDA production is probably due to the increase of SOD, GRx and GPx activities.

**Figure 8:**
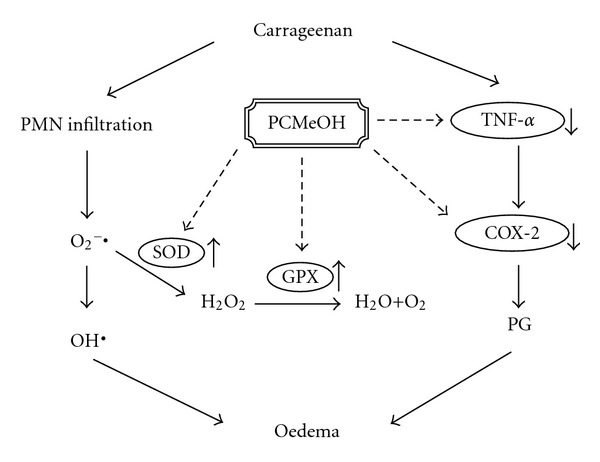
Proposed model of mechanisms in Carr-induced acute inflammatory response in the mice hind paw.

This study demonstrated that PCMeOH exhibited anti-inflammatory activity against Carr-induced paw edema and analgesic activity against nociceptive responses triggered in mice by i.p. acetic acid injection or intraplantar formalin injections. There are two possible mecha-nisms associated with the anti-inflammatory effect of PCMeOH. One is reducing the amount of AA transformed to PGs by suppressing TNF-*α* and COX-2 level. The other is cleaning away free radicals by increasing the activity of anti-oxidant enzymes, such as SOD, GRx and GPx (Figure 8). The exact mechanism by which PCMeOH exerts its analgesic effect was related to its anti-inflammatory effect, and this serves as a possible rationale for the use of PCMeOH in traditional medicine for anti-inflammation.
